# Chronic low-level expression of HIV-1 Tat promotes a neurodegenerative phenotype with aging

**DOI:** 10.1038/s41598-017-07570-5

**Published:** 2017-08-10

**Authors:** Alex M. Dickens, Seung Wan Yoo, Alfred C. Chin, Jiadi Xu, Tory P. Johnson, Amanda L. Trout, Kurt F. Hauser, Norman J. Haughey

**Affiliations:** 1 Johns Hopkins University School of Medicine Departments of Neurology, Richard T. Johnson Division of Neuroimmunology and Neurological Infections, 600N, Wolfe Street, Baltimore, MS 21287 USA; 2 Johns Hopkins University School of Medicine Department of Radiology and Radiological Science, 600N, Wolfe Street, Baltimore, MD 21287 USA; 3 Department of Pharmacology and Toxicology, Virginia Commonwealth University, Medical College of Virginia (MCV) Campus, PO Box 980613, Richmond, VA 23298-0613 USA; 4 The Johns Hopkins University School of Medicine Department of Psychiatry, 600N, Wolfe Street, Baltimore, MD 21287 USA

## Abstract

The widespread use of combinational antiretroviral therapies (cART) in developed countries has changed the course of Human Immunodeficiency Virus (HIV) infection from an almost universally fatal disease to a chronic infection for the majority of individuals. Although cART has reduced the severity of neurological damage in HIV-infected individuals, the likelihood of cognitive impairment increases with age, and duration of infection. As cART does not suppress the expression of HIV non-structural proteins, it has been proposed that a constitutive production of HIV regulatory proteins in infected brain cells may contribute to neurological damage. However, this assumption has never been experimentally tested. Here we take advantage of the leaky tetracycline promoter system in the Tat-transgenic mouse to show that a chronic very low-level expression of Tat is associated with astrocyte activation, inflammatory cytokine expression, ceramide accumulation, reductions in brain volume, synaptic, and axonal damage that occurs over a time frame of 1 year. These data suggest that a chronic low-level production of Tat may contribute to progressive neurological damage in virally suppressed HIV-infected individuals.

## Introduction

Combinational antiretroviral therapy (cART) effectively controls viral replication in the majority of HIV-infected individuals. Nevertheless, neurological manifestations of HIV infection (collectively known as HIV-associated neurocognitive disorders; HAND) continues to impact the quality of life for approximately half of HIV-infected patients^1–5^. HIV enters into the CNS early following infection^6–8^, and this CNS invasion is accompanied by structural and functional alterations that include reductions in total brain volume, thinning of cerebral cortex, and disturbances in functional connectivity^9–11^. The projected course for CNS complications in HIV infection is variable and may improve following the initiation of ART^12^, may transiently improve, persist, or worsen over time despite continued viral suppression^13–17^. These findings suggest that pharmacological control of viral replication alone is not sufficient to protect the CNS in HIV-infected individuals.

In the central nervous system (CNS), HIV infects microglia^18^, and astroglia cells^19–21^. However, production of viral particles in HIV-infected astrocytes is restricted, due to limitations in the production of HIV structural proteins^22, 23^. There is however, a chronic production of non-structural proteins Nef, Rev and Tat^23, 24^ in HIV-infected astrocytes that is thought to contribute to neuronal dysfunction^25^. Both Nef^26, 27^, and Tat^28, 29^ are known to be toxic to neurons, and Tat can be secreted from HIV-infected cells^30^, including astrocytes^31^. This chronic low-level production of Tat has been proposed to contribute to neuronal damage over prolonged periods of time, but little evidence currently exists to support this notion^32, 33^. If this assumption is correct, then current cART regimens that target HIV reverse transcriptase, protease, and viral entry^34^ would be insufficient to prevent post-integration transcription of Tat.

## Results

### Constitutive low-level tat promoter activity is associated with structural modifications in brain

**Figure 1:**
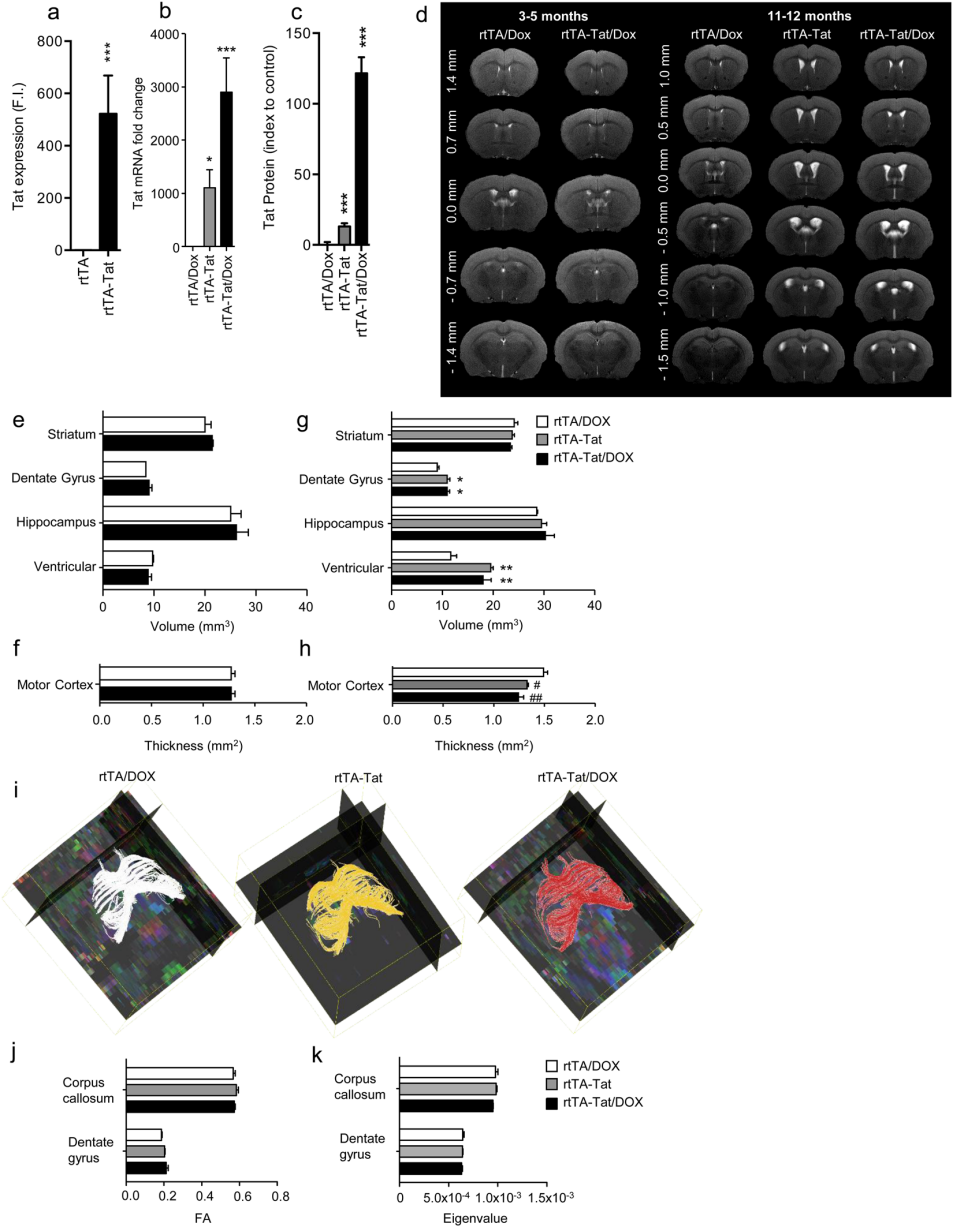
Brain volume loss is observed in doxycycline naïve rtTA-Tat mice at 12 months of age. qRT-PCR analysis showing expression of tat mRNA in cortex of (**a**) 3-month old control rtTA mice, and doxycycline naïve rtTA-Tat mice, and (**b**) 11–12 month old doxycycline treated control rtTA mice, doxycycline naïve rtTA-Tat mice, and doxycycline treated rtTA-Tat mice. (**c**) Tat protein expression in cortex of 11–12 month old doxycycline treated control rtTA mice, doxycycline naïve rtTA-Tat mice, and doxycycline treated rtTA-Tat mice. (**d**) Representative T2 weighted images from 3–5 and 11–12 month old mice of the indicated genotype and treatment conditions showing enlargement of ventricles in 11–12 month old doxycycline naïve and doxycycline treated rtTA-Tat mice compare with doxycycline treated rtTA control mice. (**e**,**f**) Bar graphs show volumetric quantification of the ventricle, hippocampus, dentate gyrus, striatum, and motor cortex of 3–5 month animals and (**g**,**h**) of 11–12 month animals. (**i**) Representative fiber tracking maps of the corpus callosum calculated from the diffusion weighted MRI images. Quantification of the fractional anisotropy (**j**) and parallel eigenvalue (**k**) the diffusion weighted images in 11–12 month old animals. Data are mean ± SEM of n = 6–7 animals/group. ANOVA with Tukey post hoc comparisons, *p < 0.05 compared to control, ***p < 0.001 compared to rtTA control.

Tetracycline-inducible transgene (rtTA) promoter systems are leaky, and exhibit chronic low-level transgene expression^32^. In 3-month old rtTA-Tat mice tat mRNA was readily detectable (Fig. 1a). In 11–12 month old doxycycline-naïve rtTA-Tat mice we found elevated levels of tat mRNA, and protein expression was 12% above background detection (Fig. 1b,c). A 21-day exposure to doxycycline resulted in a 3-fold increase in the expression of tat mRNA compared to doxycycline-naïve rtTA-Tat mice, and increased protein expression to 55% above background (Fig. 1b,c). These findings are consistent with previous results that also showed low levels of tat mRNA, and Tat protein expression in rtTA-Tat mice in the absence of doxycycline promoted *tat* gene expression^32, 35, 36^.

We first sought to determine if low-level tat gene expression resulted in time-dependent changes in brain structure using *in vivo* MRI. We did not observe changes in ventricular, striatal, hippocampal volume or motor cortical thickness of 3–5 month old doxycycline-treated rtTA-Tat mice compared with control rtTA mice (Fig. 1d–f). However, in 11–12 moth old doxycycline-naïve rtTA-Tat mice, total ventricular volume was increased from 11.43 ± 0.85 mm^3^ to 19.55 ± 0.46 mm^3^, with no further change in ventricular volume following a 21 day induction of tat gene expression with doxycycline (18.04 ± 1.57 mm^3^) (Fig. 1d,g). Primary motor cortex thickness was reduced from 1.39 ± 0.04 mm in rtTA mice, to 1.23 ± 0.03 mm in doxycycline-naïve rtTA-Tat mice, with a further small reduction to 1.19 ± 0.02 mm following doxycycline treatment (Fig. 1d,h). We also noted an increase in dentate gyrus volume from 8.99 ± 0.21 mm^3^ in rtTA mice to 10.96 ± 0.53 mm^3^ in doxycycline-naïve rtTA-Tat mice, with no further increase (10.94 ± 0.49 mm^3^) following doxycycline-induction of tat gene expression (Fig. 1d,g). We did not observe any gross alterations in the striatum or hippocampus of doxycycline-naïve or doxycycline-treated rtTA-Tat mice (Fig. 1d,g). We also did not observe any transgene-associated changes in white matter integrity or axonal damage determined by DTI using FA and parallel eigenvalues measures of the corpus callosum and dentate gyrus (Fig. 1i–k) in 11–12 month old animals.

### Chronic low-level tat expression is associated with synaptic damage

**Figure 2:**
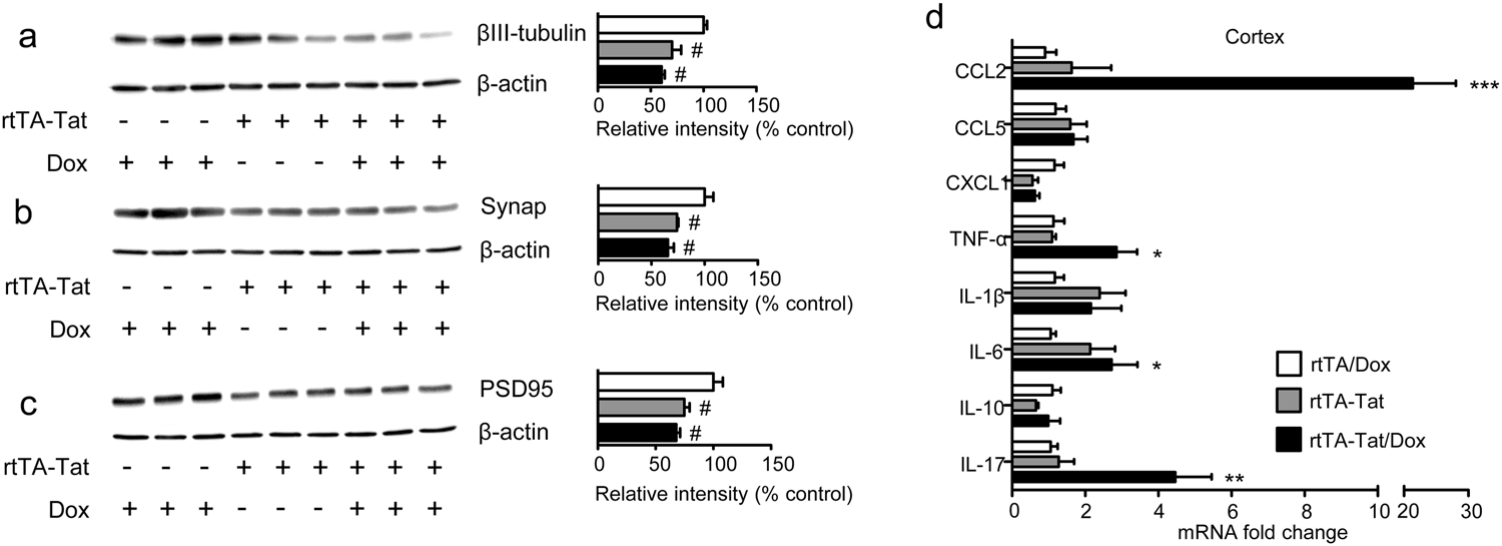
Evidence for axonal and synaptic damage in doxycycline naïve rtTA-Tat mice. Immunoblots and density quantitation of (**a**) the axonal marker βIII-tubulin, (**b**) the presynaptic marker Synaptophysin, and (**c**) the postsynaptic marker PSD95 in cortex of 11–12 month old mice with the indicated genotype and treatment condition. (**d**) qRT-PCR analysis showing expression of the indicated cytokines in cortex of 11–12 month old mice with the indicated genotype and treatment condition. Data are expressed as mean ± SEM. N = 3 mice per condition for western blots, and n = 6–7 per group for qRT-PCR. ***p < 0.001, **p < 0.01, *p < 0.05, and #*p* < 0.05 compared to rtTA control group.

We next determined if the increase of ventricular volume and reduction in cortical thickness in 11–12 month old doxycycline-naïve rtTA-Tat mice was associated with reductions in neuronal and synaptic density. Doxycycline-naïve rtTA-Tat mice exhibited a 29.8 ± 8.7% reduction in cortical levels of the neuronal specific cytoskeletal protein βIII-Tubulin, a 26.0 ± 1.3% reduction in presynaptic vesicle protein p38 (Synaptophysin), and a 25.2 ± 4.8% reduction in the postsynaptic density 95 protein (PSD-95) compared with rtTA mice, (Fig. 2a–c). Induction of *tat* gene expression did not further reduce expression of neuronal cytoskeletal or synaptic markers (Fig. 2a–c).

### Evidence for Cytokine and Cell stress in rtTA-Tat mice

**Figure 3:**
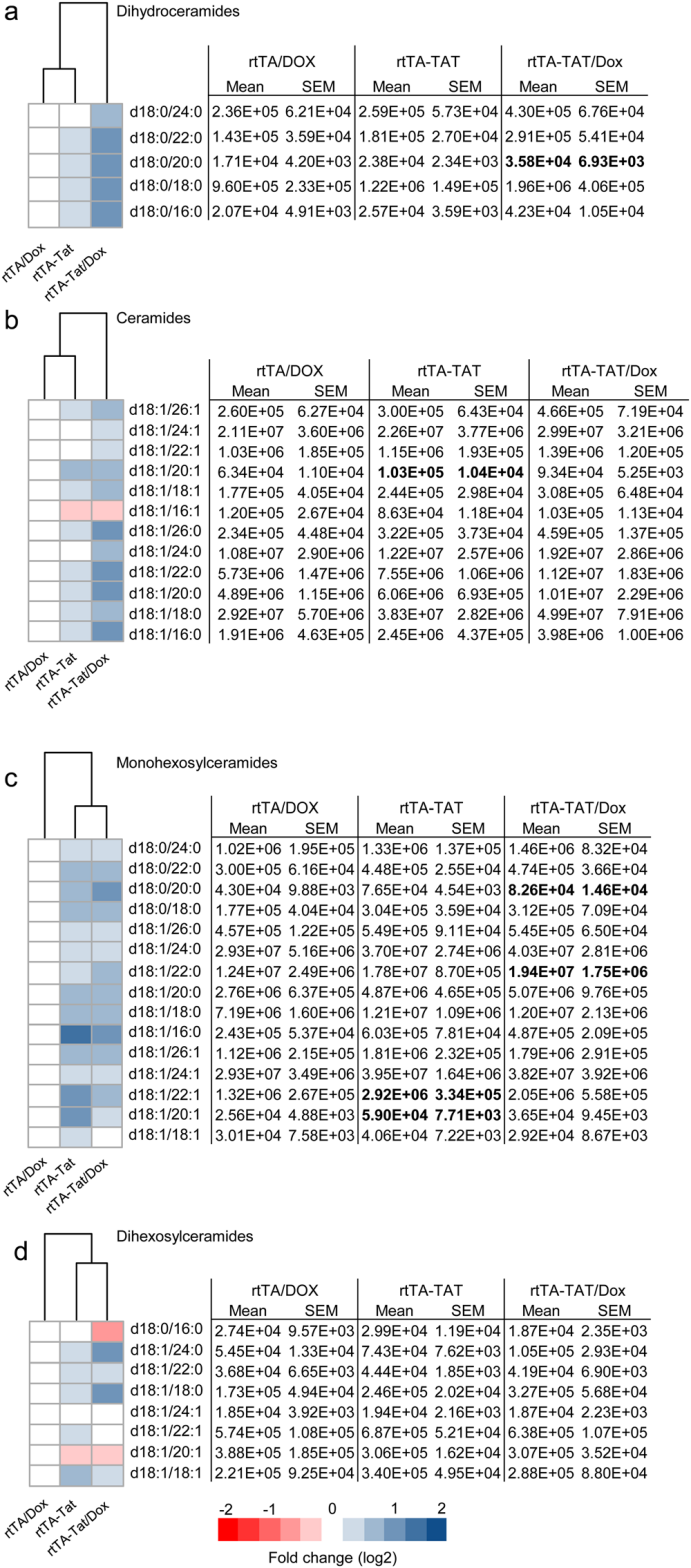
Accumulation of ceramide in cortex of rtTA-Tat mice. Concentrations of (**a**) dihydroceramides, (**b**) ceramides, (**c**) monohexosylceramides, and (**d**) dihexosylceramides, were determined in cortex of mice with the indicated genotypes. Hierarchal clustering analysis is shown to the left of each class of lipid. Blue colors indicates increase and red colors indicates decrease compared to rtTA control mice. Data are expressed as mean ± SEM, n = 6–7 per group. Bold text in table indicates *p* < 0.05 compared to rtTA control group. ANOVA with Tukey post hoc comparisons.

**Figure 4:**
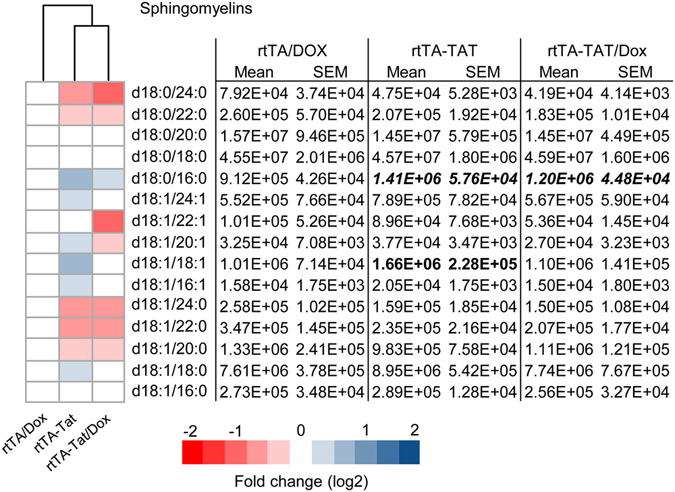
Accumulation of sphingomyelin in cortex of rtTA-Tat mice. Concentrations of sphingomyelins were determined in cortex of mice with the indicated genotypes. Hierarchal clustering analysis is shown to the left of each class of lipid. Blue colors indicates increase and red colors indicates decrease compared to rtTA control mice. Data are expressed as mean ± SEM, n = 6–7 per group. Bold text in table indicates *p* < 0.05 and italicized entries indicates *p* < 0.001 compared to rtTA control group. ANOVA with Tukey post hoc comparisons.

Expression of CCL2, CCL5, CXCL1, TNFα, IL-1β, Il-6, IL-10, IL-17 was similar in cortex of non-induced rtTA-Tat mice compared with control rtTA mice, but there were trends toward increases in in the inflammatory cytokines IL-6, and IL-1β in the non-induced rtTA-Tat mice (Fig. 2d). Induction of *tat*-gene expression resulted in significant increases of IL-6, TNFα, and the chemoattractant CCL2 (Fig. 2d). Based on previous data that HIV-1 Tat, and inflammatory cytokines can induce formation of the bioactive lipid ceramide^37–39^, we measured simple and complex ceramides in cortex and found specific accumulations of simple and complex ceramides in non-induced rtTA-Tat mice with further accumulations following induction of tat expression with doxycycline (Fig. 3a–d). Specific increases were observed with dihydroceramide C20:0, monohexosylceramide C20:1, C22:0, and C22:1. Additionally, there were accumulations of the dihydro sphingomyelin C16:0, and sphingomyelin C18:1 (Fig. 4). These data suggest that chronic low-level *tat* expression is associated with a low-level inflammatory cytokine response, and cellular stress as evidenced by accumulations of biologically active lipid species, and damage to cortical neurons.

### Increased dentate gyrus volume is associated with reactive gliosis

**Figure 5:**
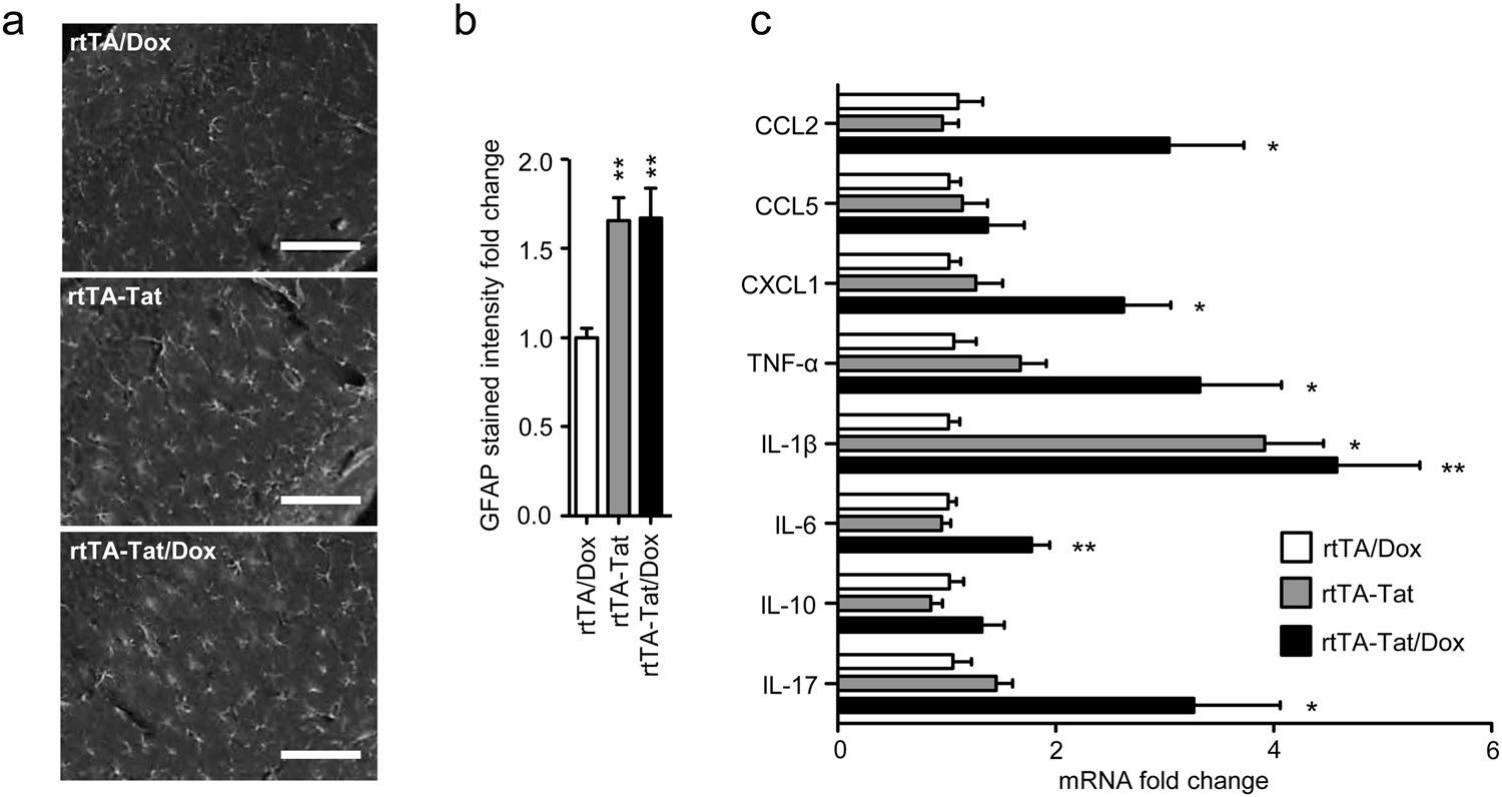
The increase in dentate gyrus volume is linked to an increase in reactive astrocytes in this brain region. (**a**) Representative images and (**b**) quantitative analysis of GFAP fluorescence from the dentate gyrus of 11–12 month old mice with the indicated genotypes. (**c**) qRT-PCR analysis showing expression of the indicated cytokines in hippocampus of 11–12 month old mice with the indicated genotype and treatment condition. Data are expressed as mean ± SEM for n = 6–7 per group. * *p* < 0.05, **p < 0.01. ANOVA with Tukey post hoc comparisons.

We reasoned that increased dentate gyrus volume in rtTA/Tat mice could be the result of enhanced neurogenesis, or edema associated with chronic inflammation. As there were no differences in the proliferation marker Ki-67 (data not shown) comparing rtTA, rtTA/Tat, and doxycycline treated rtTA/Tat mice, it is not likely that enhanced neurogenesis was responsible for the increased dentate gyrus volume in rtTA-Tat mice. The increased volume of dentate gyrus in doxycycline-naïve rtTA-Tat mice was associated with a 1.5 ± 0.1 fold increase in total area stained by GFAP, and a 1.7 ± 0.1 fold increase in GFAP intensity (a 28 day doxycycline treatment did not further increase GFAP immunoreactivity) (Fig. 5a,b). In doxycycline-naïve rtTA-Tat mice there were trends toward increases in IL-1β and IL-6 in dentate gyrus, and induction of tat gene expression resulted in significant increases in CXCL1, CCL2, IL-6, TNFα and IL-17 in dentate gyrus (Fig. 5c), suggesting that edema associated with reactive gliosis, and persistent low-level cytokine production was responsible for the increase of dentate gyrus volume seen in rtTA-Tat mice.

## Discussion

Anti-retroviral therapies have modified HIV-infection from a near universally fatal disease to a chronic infection for the majority of infected individuals. However, a variety of peripheral and central nervous system complications including impairments of cognitive function continue to be prevalent in this population^40–43^. Structural changes in brain parenchyma are apparent within the first few months following infection^9, 10, 44^, and soluble markers of immune activation, inflammation and neuronal damage are frequently detected early in the course of infection^9, 45, 46^. Initiation of cART appears to modify the trajectory of neurological damage, but does not offer complete neurologic protection or completely resolve CSF and plasma markers of inflammation, immune activation and metabolic disturbance^12, 47–49^. Structural changes in brain can persist and often worsen over time and volume reductions in multiple brain regions including grey matter, thalamus, caudate, putamen, and hippocampus have been reported^9, 10, 12, 14, 15, 44, 47, 50–53^. Likewise, functional deficits in network connectivity have been reported in older HIV patients^54^, that are only partially resolved following the initiation of cART^55^. The mechanisms for residual neurological damage in cART treated patients is not entirely clear, but it has been repeatedly proposed to involve the long-term effects of non-structural proteins in brain that continue to be expressed despite cART. However, this assumption has never been experimentally tested. Here we provide evidence for progressive brain damage when the HIV *trans*-acting protein Tat is expressed at very low-levels for prolonged periods of time.

HIV productively infects cells of macrophage lineage (including microglia)^56–58^, and produces a non-productive latent infection of astrocytes^19, 20, 59^. Under most conditions, astrocytes do not produce infectious virions, but do produce non-structural proteins Nef, Rev and Tat^23, 24^ that have been shown to be toxic to neurons in culture^26–29^. This release of neurotoxic non-structural HIV-proteins could play a significant role in neuronal damage associated with HIV infection given that up to 19% of astrocytes may be infected by HIV (estimated using highly sensitive multiplexed PCR designed detect HIV DNA in single astrocytes isolated by laser capture microdissection)^19^. The HIV-1 *trans*-activating protein Tat has received considerable attention due to the ability of this protein to be secreted from HIV-infected cells, including astrocytes^30, 31, 60^. Considering that large numbers of astrocytes could be infected^61–63^, this non-structural protein could play an important role in HIV neuropathogenesis. Tat has been shown to induce synaptodendritic damage^33, 64^, glial cell activation^65^, neuroinflammation^66^, and promotes the production of the bioactive lipid ceramide^67^. Mechanisms for Tat-induced neuronal damage appears to involve a rapid stimulation of endoplasmic reticulum calcium release, NMDA receptor calcium leak, enhanced agonist-evoked NMDA receptor channel function, and endolysosomal stress^29, 68–70^. In rtTA-Tat mice that express Tat under the control of a tetracycline-inducible GFAP promoter, doxycycline-induced transgene expression results in impairments of reference and working memory^33, 71^, with no apparent deficits in sensorimotor function or general activity^33^. These behavioral changes were associated with reductions in cortical volume^72^, dendritic pruning, and synaptic loss in the cortex and hippocampus^36, 71, 73^. In these published studies, a robust expression of Tat was induced by the administration of doxycycline to turn on Tat expression under the control of a tetracycline-inducible GFAP promoter system. In the present study, we took advantage of a known defect in the tetracycline inducible gene system that allows a “leaky” transgene expression in the absence of doxycycline to study the effects of chronic low-level Tat expression on brain structure. We found that low-level chronic expression of Tat was associated with reductions in brain volume (increased ventricular volume), and motor cortex in 11–12 month old mice, but not in 3–5 month-old mice. These gross pathological changes were accompanied by axonal and synaptic loss in 11–12 month old non-induced rtTA-Tat mice. These data suggest that chronic low-level expression of Tat was sufficient to reduce brain volume and injure synapses over the time frame of 11–12 months.

Reductions in brain volume and neuronal damage in non-induced rtTA-Tat mice may be related to a chronic low-level inflammatory response. In non-induced rtTA-Tat mice, we found evidence for astrocyte activation, low-level inflammatory cytokine expression, and accumulation of the stress-induced bioactive lipid ceramide, each which have been shown to be induced by Tat and to damage neurons in culture^37, 74, 75^. We observed a general increase in astrocyte activation in non-induced rtTA-Tat mice as measured by GFAP expression that was not substantially increased following induction of Tat expression with doxycycline. We also observed a general trend towards increases of CCL2, IL-1, and IL-6 in the cortex of non-induced rtTA-Tat mice with elevations of CCL2, TNFα, IL-6 and IL-17 following doxycycline induction of Tat expression. In hippocampus, we found a selective increase in the expression of IL-1β in non-induced rtTA-Tat mice that was accompanied by increased expression of CCL2, CXCL1, TNFα, IL-6 and IL-17 following doxycycline induction of Tat expression. While the induction of cytokine expression by Tat has been documented^76–78^, this selective increase of IL-1β expression resulting from a long–term exposure to low levels of Tat is unusual, and may be associate with the presence of a focal edema in hippocampus. Data from MRI studies in Multiple Sclerosis that have demonstrated an increase in brain volume during active inflammation supports this notion^79–81^.

In addition to inducing Tat mRNA expression in GFAP+ brain astroglia, Tat mRNA is also induced in GFAP+ myenteric glia within 72 h, and reduces gut microbiota^82^. If doxycycline is withheld, Tat continues to be expressed at 3 weeks and commensal gut bacteria recolonize within 2 weeks^83^. Upon microbial recolonization in the absence of doxycycline, Tat heightens gut-barrier leakiness resulting in increased levels of serum LPS, proinflammatory cytokines in the gut, and bacteria in the mesenteric lymph nodes, spleen, and liver^83^, which could potentially influence peripheral immune response, CNS pathology and function^84^. The interactions of HIV-Tat with gut epithelia, immune/inflammatory responses, and effects on CNS function warrant further consideration.

We did not observe changes in white matter structure of non-induced or doxycycline-induced rtTA-Tat mice as determined by DTI imaging. It has previously been reported that the induction of Tat expression reduces cortical gray matter density in young Tat transgenic mice^72^, and modifies the structure of myelin observed by either electron microscopy^85^, or DTI imaging^86^ with reductions of fractional anisotropy and behavioral changes. There are several differences between these studies and our own that could account for these discrepancies. A previously reported DTI study^86^ was performed *ex-vivo* which allows for longer scan times, lower background noise, and higher resolution imaging that may have been more sensitive to reliably detect changes in white matter structure. Likewise, EM is more sensitive than *in vivo* DTI to detect changes in white matter structure. In addition, doxycycline induction was accomplished by intraperitoneal administration at the dose of 100 mg/kg/day for 7 days for the DTI study^86^, and chow containing 6 g/kg starting at 3 months of age for a 12-week period for the EM study^85^, compared to our protocol where doxycycline was administered at the dose of 2 mg/mL in drinking water containing 0.8% sucrose for 21 days. It is possible these differences in route of administration, dose, and duration of dosing could account for these experimental differences in the presence or absence of white matter damage. By contrast, another report (using the same line of Tat transgenic mice and IP doxycycline injections) found no changes in striatal volume using stereology (similar to the present findings) despite a significant loss of dendrites and dendritic spines by striatal neurons following 3 months of Tat induction (doxycycline 6 mg/g chow)^85^.

These data demonstrate that even very low-level expression of Tat in brain can result in chronic glial activation, cytokine expression, and structural damage over prolonged time periods of time. These findings suggest that current cART regimens may not be sufficient to protect the brain from the effects of non-structural proteins that continue to be expressed despite suppression of viral replication.

## Methods

### Animals

Tetracycline-inducible glial fibrillary acidic protein (GFAP)-driven HIV-1 Tat transgenic mice (rtTA-Tat) from two age groups (3–5 months, and 11–12 months) and aged matched control mice expressing only the rtTA promoter were used for this study^73^. Induction of tat gene expression was achieved by the addition of doxycycline (2 mg/mL) in drinking water containing 0.8% sucrose for 21 days. Non-induced mice received 0.8% sucrose in water as a vehicle control. Water bottles were wrapped in tin foil to prevent breakdown of doxycycline by light, and water was refreshed every two days. All experimental protocols were approved by the Johns Hopkins University School of Medicine Animal Care and Use Committee and were conduced in accordance with guidelines for the ethical treatment of animals.

### Magnetic resonance imaging

MRI experiments were performed on a horizontal bore 11.7 T Bruker Biospec system (Bruker, Ettlingen, Germany). A 72 mm quadrature volume resonator was used as transmitter, and a 2 × 2 mouse phased array coil was used for image acquisition. Brain volume measures were performed *in vivo* using a rapid acquisition with refocused echoes (RARE) sequence with the following parameters: repetition time (TR), 5 s; effective echo time (TE), 59 ms; field of view (FOV), 15 × 15 mm^2^; image matrix, 196 × 196; slice thickness (SI), 0.5 mm; number of average (NA), 4; pixel resolution, 0.0765 × 0.0765 mm^2^. In order to align the T2-weighted and DTI images, a sagittal T2-weighted image was first acquired. The center slice was aligned to the anterior commissure, and the slices were offset by 1.5 mm (anterior) to ensure whole brain coverage with 35 MRI slices. Animals were anesthetized using 2% isoflurane, and anesthesia maintained by 1% to 1.5% isoflurane during the MRI scan. Mice were placed on a water-heated bed equipped with temperature and respiratory control. Volumetric quantification of ventricles, striatum, hippocampus, dentate gyrus region and motor cortex were conducted by manual tracing region of interests (MRI Studio, Center for Imaging Sciences, Johns Hopkins University), using the Allen mouse brain atlas as a reference. For striatal tracings, the inferior striatal boundary was defined as the intersection between two lines: one line drawn through the rhinal fissure and tangent to the bottom side of the anterior commissure, and the second line drawn through the notch of the olfactory tract tangent to the topside of the anterior commissure^87^. The dentate gyrus region was defined as the area under the hippocampal fissure. Motor cortical thickness was defined as the distance between the apex of the corpus callosum to the surface of the cortex vertically above Bregma. Thickness measurements were conducted in both hemispheres of the brain and the resulting data averaged.

### Diffusion Tensor Imaging

Multi-slice Diffusion Tensor Imaging (DTI) of adult mice was performed using a 4-segment diffusion weighted EPI sequence with the following parameters: TE/TR = 23/9000 ms, 1 NA, 30 diffusion directions, *b* = 1500 s/mm^2^, with a partial Fourier factor of 1.3 in the phase encoding. FOV, slice thickness, in plane resolution, and number of slices were identical to parameters used for T_2_-weighted images. Including respiratory gating, total imaging time was approximately 20 minutes. Second order shimming on the whole brain was performed before DTI imaging.

### Quantitative Real-Time PCR (qRT-PCR)

Tat and cytokine mRNA levels were measured by qRT-PCR. Fresh cerebral cortex was flash frozen and RNA isolated using the RNeasy kit (Qiagen). RNA (1 µg) was diluted to a final volume of 10 µl in DEPC water prior to the addition of 1 µl of random primers (Invitrogen) and 1 µl of dNTP’s (10 mM; Invitrogen). The resultant mixture was incubated at 65 °C for 5 min, and then stored on ice. To this mixture, 8 µl of master mix, 4 μl of 5x first strand buffer (Invitrogen), 2 μl of 0.1 M DTT (Invitrogen), 1 μl of RNasin (Promega), and 1 μl of Superscript II RT (18064, Invitrogen) were added and the mixture incubated at room temperature for 10 min, 42 °C for 50 min, and 70 °C for a further 15 min. The cDNA was stored at −20 °C until required for qRT-PCR. cDNA (1 µl) was mixed with 12.5 µl of SYBER Green Master Mix (Applied Biosystems), 10.5 μl of DEPC water, and 0.5 µl of the requisite forward and backward primers to amplify CCL5, CXCL1, IL-10, IL-17, IL-1β, IL-6, TNF-α, and CCL2 (Sigma Aldrich). Each sample was run as duplicate and each plate included a non-template control to normalize plate-plate variability. Amplification was conducted using 7300 qRT-PCR instrument (Applied Biosystems) with the following parameters: 1 cycle at 50 °C for 2 min, 1 cycle at 95 °C for 10 min, 40 cycles of 95 °C for 15 s and 60 °C for 1 min, and for the dissociation stage 95 °C for 15 s, 60 °C for 1 min and 95 °C for 15 s. The change in threshold cycle (ΔCT) was then normalized for each sample to β-actin. The ΔΔCT values for each sample was generated using the average ΔCT for the rtTA/DOX group. The ΔΔCT data was then linearized (2^−ΔΔCT^) resulting in the data being expressed as a fold change relative to the control animals (rtTA/DOX).

### Immunohistochemistry

Mice were transcardially perfused, brains were rapidly removed, and separated into halves with a coronal cut. One half of the brain was fixed for 24 h with 4% paraformaldehyde, followed by cryoprotection in 30% sucrose (v/v, Sigma), and the second half was flash frozen. Microtome sections (40 μm; HM450, Mikron Instruments) were cut and stored at −20 °C in 30% sucrose, 30% ethylene glycol, and 0.05 M phosphate-buffered saline. A primary antibody directed against GFAP (1:1000, Sigma) was applied overnight at 4 °C in Tris-buffered saline (TBS; 100 mM Tris-Cl, pH 7.5, 150 mM NaCl) containing 0.1% Tween-20 and 10% normal goat serum. Sections were washed 3 × 10 min in TBS, and secondary antibody (1:1000, goat anti-Rb IgG Alexa 594; company; Invitrogen) was applied for 1 h in dark In TBS containing 5% normal goat serum. Sections were washed 3 × 10 min in TBS, loaded onto slides with Vectashield Antifade Mounting Medium containing DAPI (Vector Laboratories). For each brain, microscopic images of CA1, CA2/CA3, CA3/dentate gyrus, and dentate gyrus at were acquired for six consecutive sections spaced 40 µm apart using a 10X objective mounted on an automated Eclipse 90i microscope equipped with NIS-Elements software (Nikon Instruments). GFAP-stained area and the intensity of the thresholded area were summed for CA1, CA2/CA3, CA3/dentate gyrus, and dentate gyrus for each section and expressed as the percentage of ROI area.

### Immunoblot analysis

Fresh frozen cerebral cortex was homogenized in RIPA buffer (50 mM Tris-Cl, pH 7.5, 150 mM NaCl, 10 mM EDTA, 2 mM EGTA, 50 mM NaF, 0.5% SDS, 1% NP-40) containing a protease inhibitor cocktail (Roche Applied Science). Protein concentrations were determined by BCA (Thermo Scientific). Proteins were separated on an SDS-PAGE gel, and transferred to a nitrocellulose membrane (Bio-Rad). Membranes were blocked in TBS (100 mM Tris-Cl, pH 7.5, 150 mM NaCl) containing 0.1% Tween-20, and 5% nonfat milk solution for 1 h, then incubated with one of the following primary antibodies: mouse-anti-PSD95 (1:1000; EMD Biosciences), mouse-anti-synaptophysin (1:500; Sigma), or mouse anti-βIII-Tubulin (1:2000; Sigma) overnight at 4 °C on a rotary shaker. Membranes were washed three times with 0.1% TBS prior to addition of an anti-mouse IgG horse radish peroxidase secondary antibody (1:2000; Cell Signaling Technology). Proteins were visualized by chemiluminescence (Millipore) on a QBOX imaging system (Syngene). Densitometry analysis was performed in ImageJ (NIH) using the gel analyzer plug-in.

### Tat ELISA

Brain tissues were homogenized in NP40 lysis buffer supplemented with protease and phosphatase inhibitors using a rotor-stator homogenizer. Protein was isolated and quantified by bicinchoninic acid assay. ELISA was performed as described previously^88^ briefly 96-well plate was coated with 0.2 ug of mouse anti-Tat (Biolegend # 919001) per well. After washing and blocking samples and standards were diluted in 2.5% (v/v) non-fat milk in PBST and incubated overnight at 4 °C. Following washing 0.45 ug of rabbit anti-Tat biotinylated antibody was incubated for one hour at room temperature per well (Abcam ab43015). Signal was amplified with strepavidin-HRP and developed colorimetrically.

### Mass Spectrometry

A crude lipid extraction was performed from fresh frozen cerebral cortex using a modified Bligh and Dyer procedure as previously described^67, 89^. Lipid species were separated by gradient elution using high performance liquid chromatography (HPLC; Shimadzu) C18:0 reverse-phase column (Phenominex). As sample eluted of the column it was introduced into an electrospray ion source and lipid species were identified by multiple reaction monitoring on two different LC/ESI/MS/MS systems (an API3000 for detection of ceramides and a 4000 QTrap for sphingomyelins; both from ABSciex) using instrument parameters previously described^90, 91^. Instruments were controlled by Analyst 1.4.2 (API3000), and Analyst 1.5.1 (4000 Q Trap). Area under the curve for each peak was calculated by MultiQuant (ABSciex). The following classes of lipid were identified: dihydroceramide, ceramide, monohexosyl-, dihexosyl-ceramide and sphingomyelin with acyl chain lengths 16:0–26:1.

### Statistical Analysis

Figures and statistical analyses were generated using Prism (Version 6 GraphPad). Statistical comparisons were conducted by ANOVA with Tukey post hoc tests. Hierarchal clustering of lipid data and heatmaps were created using the pheatmap package in R software (pheatmap version 0.7.7, R version 3.1.1).
